# Prevalence, socio-economic, and associated risk factors of oral cavity parasites in children with intellectual disability from Lorestan province, Iran

**DOI:** 10.3389/fcimb.2024.1398446

**Published:** 2024-06-20

**Authors:** Behnoush Selahbarzin, Hossein Mahmoudvand, Amal Khudair Khalaf, Fahimeh Kooshki, Fatemeh Farhadi, Parastoo Baharvand

**Affiliations:** ^1^ Nutritional Health Research Center, Lorestan University of Medical Sciences, Khorramabad, Iran; ^2^ Department of Pediatric Dentistry, School of Dentistry, Lorestan University of Medical Sciences, Khorramabad, Iran; ^3^ Razi Herbal Medicines Research Center, Lorestan University of Medical Sciences, Khorramabad, Iran; ^4^ Department of Microbiology, College of Medicine, University of Thi-qar, Thi-qar, Iraq; ^5^ Department of Pediatric Dentistry, School of Dentistry, Shahid Beheshti University of Medical Sciences, Tehran, Iran; ^6^ Student Research Committee, Lorestan University of Medical Sciences, Khorramabad, Iran; ^7^ Department of Community Medicine, Lorestan University of Medical Sciences, Khorramabad, Iran

**Keywords:** parasites, children, prevalence, Iran, oral cavity

## Abstract

**Introduction:**

Children with intellectual disability (ID) often face challenges in maintaining proper oral hygiene due to their motor, sensory, and intellectual impairments, which can lead to compromised oral health; therefore, there is a need to enhance the oral health status of these populations and establish an effective system for administering preventive interventions. Here, we aimed to evaluate the prevalence of *Entamoeba gingivalis* and *Trichomonas tenax* among children with ID in Lorestan province, in Western Iran through parasitological and molecular methods.

**Methods:**

The current descriptive investigation involved 215 in children with ID and 215 healthy children (non-ID) who were referred to health facilities in Lorestan province, Iran between October 2022 and March 2024. The prevalence of protozoa in the oral cavity was found through the utilization of both microscopic analysis and conventional polymerase chain reaction (PCR) techniques.

**Results:**

The total prevalence of the *E. gingivalis* and *T. tenax* in children with ID was found to be 87 (40.5%) and 92 (42.8%) through microscopic and PCR methods, respectively. Among the positive samples, 57 (61.9%) and 35 (38.1%) children tested positive for *E. gingivalis* and *T. tenax*, respectively. In contrast, among the 215 non-ID children in the control group, 39 (18.1%) and 42 (19.5%) tested positive by microscopic and PCR methods, respectively. Among positive samples in non-ID children, 23 (54.7%) and 19 (45.3%) children were positive for *E. gingivalis* and *T. tenax*, respectively. Multiple logistic regression analysis indicated that residing in urban areas, parental education, monthly family income, and tooth brushing p<0.001) were identified as independent risk factors for oral cavity parasites.

**Conclusion:**

This study identified a notable prevalence of oral cavity parasites in children with ID in Lorestan province, Western Iran. It is imperative to recognize the primary risk factors associated with these parasites, particularly inadequate teeth brushing, in order to enhance public and oral health strategies for children with ID. Therefore, pediatric dental professionals should remain vigilant regarding these risk factors to effectively recognize and address oral health issues in this population, thereby mitigating the occurrence of oral diseases and infections.

## 1 Introduction

Oral and dental hygiene are crucial factors in maintaining overall health and well-being. Neglecting basic oral care practices not only harms the teeth and gums but also significantly elevates the risk of heart disease, cancer, and diabetes (Dörfer et al., 2017). The oral cavity, along with the teeth, serves as the primary digestive organ in the human body. From a scientific perspective, the initial breakdown of food commences in the mouth, facilitated by oral enzymes and tooth movement (Gift and Atchison, 1995). Given these insights, any malfunctions in the oral cavity and teeth can lead to severe digestive and functional complications (Wu et al., 2016). Thus, the maintenance of oral and dental hygiene is crucial and can potentially lead to significant systemic issues (Wu et al., 2016). Data from the World Health Organization reveals that despite the emphasis on oral health, a significant portion of the global population, especially children, struggle with dental and gum diseases, a predicament that is widespread among underprivileged segments of society (Sadida et al., 2021).

Maintaining optimal oral health is a crucial component of overall well-being, particularly for children, and holds even greater significance for those with special health requirements (Kilian et al., 2016). Despite the heightened prevalence of dental issues among individuals with disabilities or illnesses, they often receive inadequate oral care compared to the general population (Mishu et al., 2022). Researches indicated that dental treatment stands as the most neglected health necessity among disabled individuals. Therefore, the principal objective of dental care provision for this demographic should prioritize preventive measures to address dental diseases, necessitating meticulous planning and effective service delivery (Peres et al., 2019). Intellectual disability (ID) is a genetic condition characterized by substantially below-average intellectual functioning and deficiencies in adaptive behavior (Chelly et al., 2006). This condition typically emerges in childhood and is distinguished by diminished intelligence and adaptive skills (Chelly et al., 2006).

*Entamoeba gingivalis* and *Trichomonas tenax* are anaerobic parasites found in the human oral cavity (Bonner et al., 2014; Marty et al., 2017). These parasites can be transmitted through saliva, contaminated food containers, drinking water, and other means (Bonner et al., 2014; Marty et al., 2017). They typically inhabit the upper regions of the digestive system, including the mouth, teeth, gum margins, and spaces between teeth, as well as the respiratory tract (Arpağ and Kaya, 2020; Eslahi et al., 2021). While traditionally considered benign microorganisms by many dentists, recent studies have linked these parasites to oral conditions like periodontal disease, gingivitis, and osteomyelitis (Bao et al., 2020; Yaseen et al., 2021; Azadbakht et al., 2022). Children with ID often face challenges in maintaining proper oral hygiene due to their motor, sensory, and intellectual impairments, which can lead to compromised oral health; therefore, there is a need to enhance the oral health status of these populations and establish an effective system for administering preventive interventions. Here, we aimed to evaluate the prevalence of *E. gingivalis* and *T. tenax* among children with ID in Lorestan province, in Western Iran through parasitological and molecular methods.

## 2 Materials and methods

### 2.1 Study area

**Figure 1:**
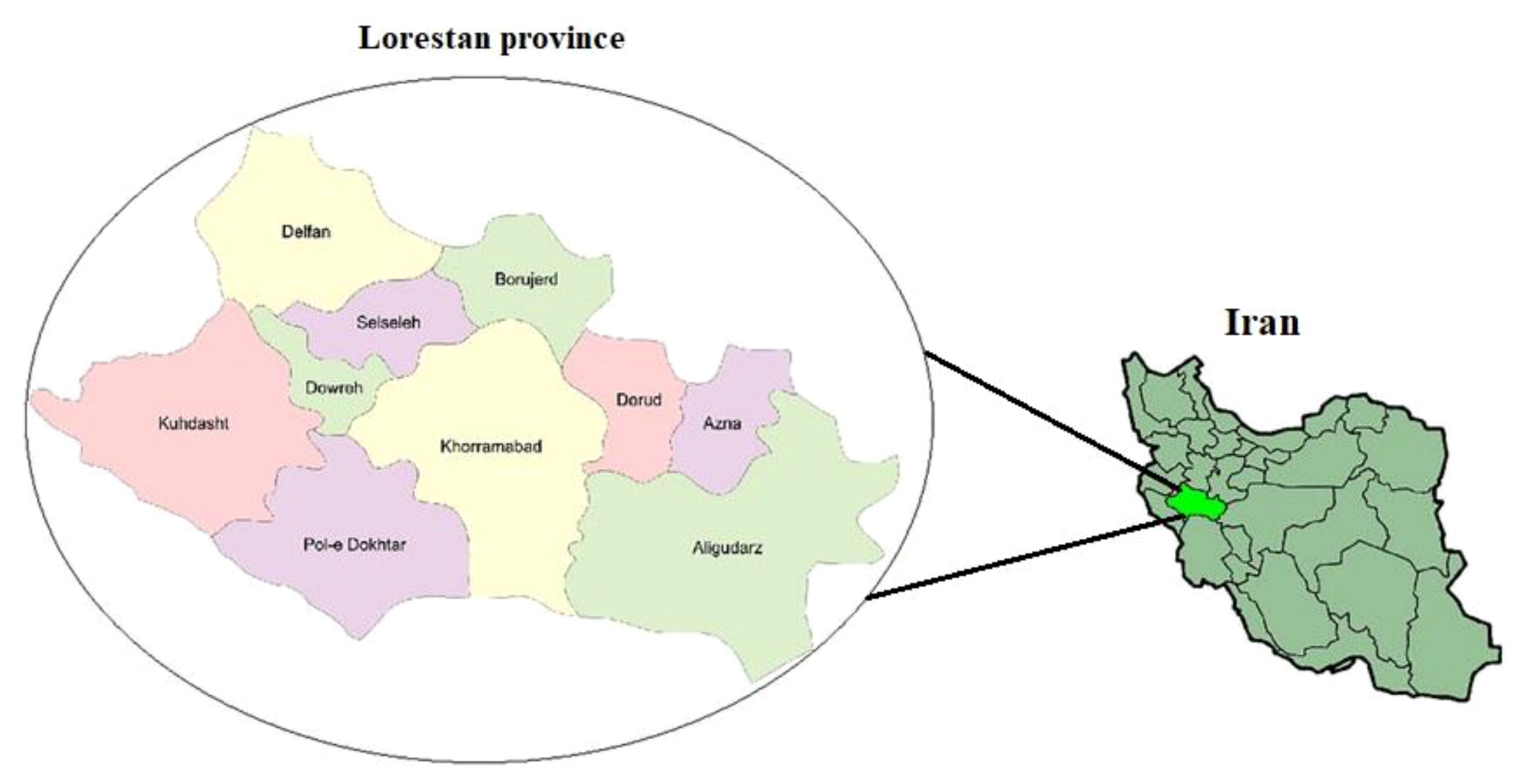
Geographical location of the study area, where the study was carried out.

The Lorestan province, located in the western region of Iran, shares borders with the provinces of Isfahan, Kermanshah, Markazi, Khuzestan, Hamedan, and Ilam. Encompassing an area of approximately 28,000 square kilometers, the province is home to a population of nearly 2 million individuals (**Figure 1**).

### 2.2 Participants

The current descriptive investigation involved 215 ID children who were referred to health facilities in Lorestan province, Iran between October 2022 and March 2024. Additionally, a control group consisting of 215 healthy children without ID (non- ID) who were referred to health centers during the same study period was included. Exclusion criteria encompassed refusal to participate in the study, recent use of systemic antibiotics within the past three months, and immunocompromised status.

### 2.3 Questionnaire, socio-economic, and the possible risk factors

Information and consent documents were created and distributed to the parents of participants in both experimental groups. Subsequently, a questionnaire was administered to gather demographic data such as age, gender, place of residence, parental education level, head of household’s occupation, monthly family income, toothbrushing habits, and mouthwash usage. The questionnaire was completed with the assistance of the participants prior to data collection.

### 2.4 Collecting samples

Specimens were collected from each child for microscopic evaluation by obtaining two samples using sterile swabs from saliva and dental plaques. Additionally, a third sample was placed in tubes with sterile normal saline for molecular testing purposes (Azadbakht et al., 2022).

### 2.5 Parasitological examination

Following the preparation of smears on glass slides, the slides underwent staining procedures using trichrome and Giemsa stains, and were subsequently examined using a light microscope (Alam-Eldin and Abdulaziz, 2015).

### 2.6 DNA purification

DNA purification of the saliva/dental plaque samples was carried out using commercial kit (Qiagen, Germany) according to the producer’s instruction.

### 2.7 Amplification and conventional polymerase chain reaction

The extracted DNAs were used for conventional PCR analysis for identification of oral parasites. Two distinct sets of PCR primers were employed to amplify and for the detection of *E. gingivalis* and *T. tenax* as previously described by Azadbakht et al. (2022) including: SrRNA gene for *E. gingivalis* using the primers of Forward: 5′- GCGCATTTCGAACAGGAATGTAGA -3′ and Reveres: 5′-CAAAGCCTTTTCAATAGTATCTTCATTCA-3′, as well as 18S ribosomal RNA gene for *T. tenax* using the primers of forward (5′-ATGACCAGTTCCATCGATGCCATTC-3′) and reverse (5′-CTCCAAAGATTCTGCCACTAACAAG -3′). The size of the PCR bands for the *E. gingivalis* and *T. tenax* was 454 and 496 bp, respectively. The PCR thermal procedure involved an initial denaturation step lasting 6 minutes at 94°C, followed by 35 cycles of denaturation at 94°C for 30s, primer annealing at 60°C for 1 minute, and elongation at 72°C for 1 minute. A final elongation step was conducted for 10 minutes at 72°C (Azadbakht et al., 2022). Appropriate positive and negative controls were employed to validate the quality of DNA extraction and PCR outcomes and to eliminate the possibility of contamination. Positive controls included patient samples containing motile *E. gingivalis* and *T. tenax* that produced the anticipated amplicon sizes, while nuclease-free water served as the negative control. The amplicons were visualized by agarose gel (1%) electrophoresis.

### 2.8 Statistical analysis

Following the acquisition of necessary data, descriptive statistics were employed to elucidate the dataset. The relationship between the variables of interest and the prevalence of oral cavity protozoa was assessed through Chi-square and Fisher exact tests. Additionally, logistic regression was used to calculate the odds ratio along with its 95% confidence intervals. These statistical analyses were conducted using SPSS version 25 software, with statistical significance set at a threshold of P < 0.05.

## 3 Results

### 3.1 Participants

In the present case-control investigation, totally, 430 participants including 215 children with ID and 215 non-ID children referred to health facilities of Lorestan Province, Iran, were studied to evaluate the prevalence of oral cavity parasites (**Table 1**). The mean age of the participants in the ID and non-ID groups was 10.6 ± 2.21 and 11.1 ± 3.41 years, respectively. The majority of participants were male in the ID (122, 56.7%) and non-ID (137, 63.7%) groups. In terms of residence, 137 (63.7%) and 128 (59.5%) participants in the ID and non-ID groups lived in urban areas, respectively, and the rest lived in rural parts. Among the participants in the ID and non-ID groups, 154 (71.6%) and 97 (45.1%) children had parents with degrees lower than diploma, respectively. This difference was statistically significant between the groups (p < 0.001). Moreover, in 53 (22.6%) of participants in the ID group and 69 (33.1%) in the non-ID group, the head of the household was an employee. Between the children in the ID and non-ID groups, 74 (34.4%) and 126 (58.6%) brushed their teeth daily, respectively. Considering the use of mouthwash, 17 (7.0%) and 39 (18.2%) children in the ID and non-ID groups used the mouthwash, respectively. This difference was statistically significant between the groups (p<0.001) (**Table 1**).

**Table 1:** Demographic, socio-economic, and risk factors of participants in children with intellectual disability (ID) and non- intellectual disability children (non-ID) groups.

Variable	Group		P value
	ID No. (%)	Non-ID No. (%)	
Age			
≥10 yrs	158 (73.5)	145 (67.4)	0.205
<10 yrs	57 (26.5)	70 (32.6)	
Gender			
Male	122 (56.7)	137 (63.7)	0.168
Female	93 (43.3)	78 (36.3)	
Residence			
Rural	78 (36.3)	87 (40.5)	0.393
Urban	137 (63.7)	128 (59.5)	
Parent education			
<Diploma	154 (71.6)	97 (45.1)	0.001*
≥Diploma	61 (28.4)	118 (54.9)	
Head of household’s occupation			
Unemployed	18 (8.4)	13 (6.1)	0.276
Employee	53 (22.6)	69 (33.1)	
Freelancer jobs	144 (67.0)	133 (61.8)	
Monthly family income			
≤200 USD	112 (56.7)	119 (55.3)	0.374
200–600	87 (40.5)	70 (32.6)	
≥600 USD	16 (7.4)	26 (12.1)	
Tooth brushing			
No	141 (65.6)	89 (41.4)	0.001*
Yes	74 (34.4)	126 (58.6)	
Mouthwash			
No	200 (93.0)	175 (81.4)	0.001*
Yes	15 (7.0)	40 (18.6)	

*p < 0.05, difference was statistically significant.

### 3.2 Prevalence of oral cavity protozoan parasites

**Figure 2:**
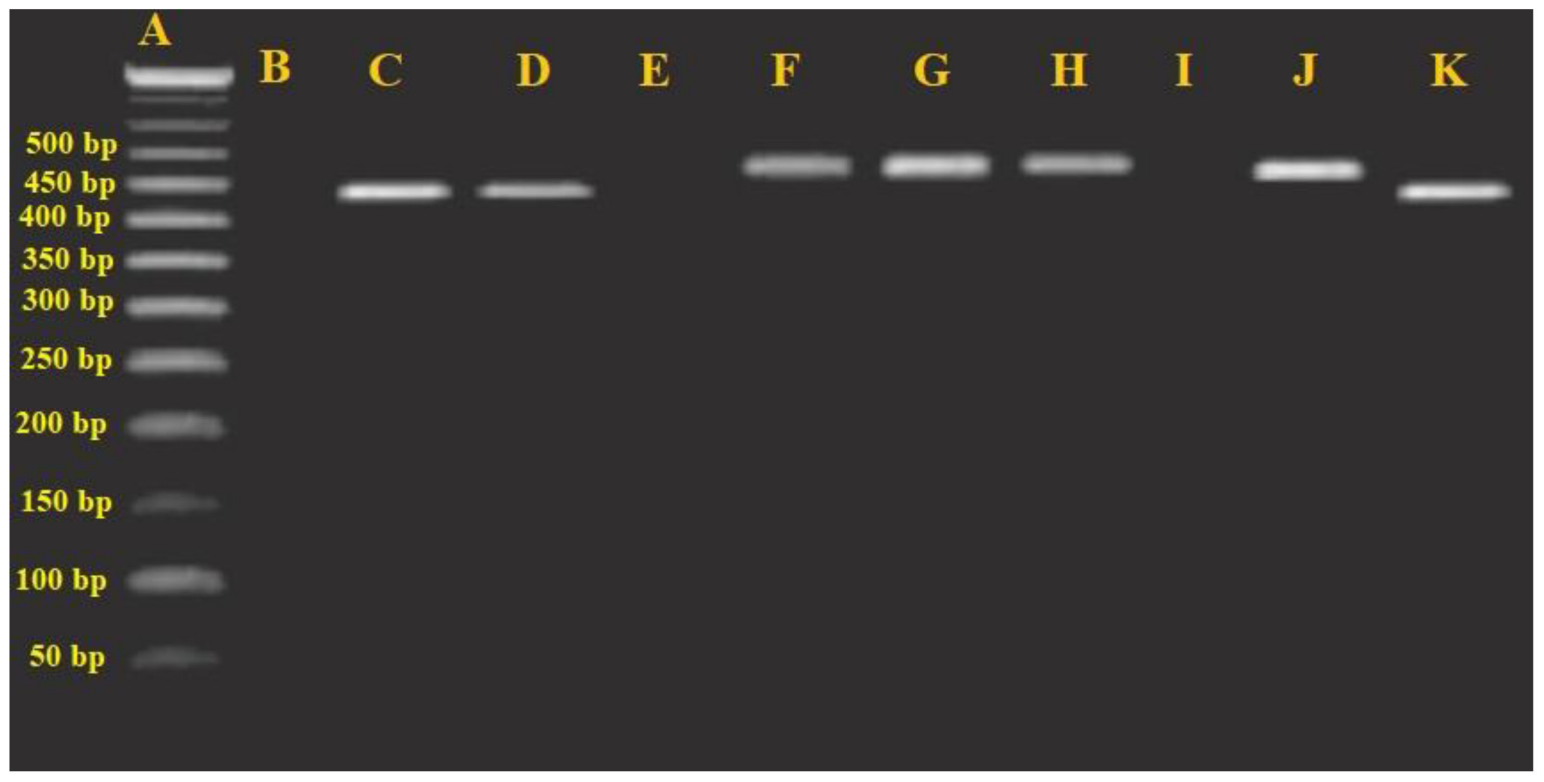
Agarose gel electrophoresis analysis of the PCR products. **(A)** Ladder (50 bp); **(B)** negative control; **(E, I)** negative samples; **(C)** *Entamoeba gingivalis* positive control, 454 bp; **(D, K)** *E*. *gingivalis* positive sample; **(F)** *T. tenax* positive control, 496 bp; **(G, H, J)** and **(J)** *T. tenax* positive sample.

The total prevalence of the *E. gingivalis* and *T. tenax* in children with ID was found to be 87 (40.5%) and 92 (42.8%) through microscopic and PCR methods, respectively (**Figure 2**), respectively. Among the positive samples, 57 (61.9%) and 35 (38.1%) children tested positive for *E. gingivalis* and *T. tenax*, respectively. In contrast, among the 215 non-ID children in the control group, 39 (18.1%) and 42 (19.5%) tested positive by microscopic and PCR methods, respectively. Among positive samples in non-ID children, 23 (54.7%) and 19 (45.3%) children were positive for *E. gingivalis* and *T. tenax*, respectively (**Table 2**). The findings indicate that the likelihood of being exposed to oral cavity parasites in non-ID children was significantly lower compared to the case group (p<0.001, OR = 0.325; CI= 0.211–0.500).

**Table 2:** Comparison the prevalence of oral cavity parasites in children with intellectual disability (ID) and non- intellectual disability children (non-ID) groups.

Group	Microscopic test		PCR method		OR	CI	P value
	Positive No. (%)	Negative No. (%)	Positive No. (%)	Negative No. (%)			
ID	87 (40.5)	128 (59.5)	92 (72.8)	123 (272)	0.325	0.211–0.500	<0.001^*^
Non-ID	39 (18.1)	176 (81.9)	42 (19.5)	173 (80.5)	–	–	–

*p < 0.05, difference was statistically significant. -, Not determined.

### 3.3 Socio-economic and related risk factors

In the investigation of age-related subgroups, no statistically significant correlation was found between the occurrence of both oral cavity parasites and the age of participants in both the ID (p=0.172) and non-ID groups (p=0.918). Similarly, no significant association was observed between gender and the prevalence of both oral cavity parasites among participants in the ID (p=0.437) and non-ID groups (p=0.279). However, a notable relationship was identified between the participants’ place of residence and the prevalence of both oral cavity parasites in both the ID (p=0.008) and non-ID groups (p<0.001).

In terms of socio-economic factors, a significant correlation was identified between monthly family income and the prevalence of oral cavity parasites in both the ID (p<0.001) and non-ID groups (p<0.001). Conversely, there was no notable association between parental education level and the prevalence of both *E. gingivalis* and *T. tenax* in the ID group (p=0.975) or the non-ID group (p=0.586). Similarly, no significant correlation was found between the head of the household’s occupation and the prevalence of both *E. gingivalis* and *T. tenax* in both the ID (p=0.612) and non-ID groups (p=0.312). Analysis of tooth brushing habits revealed a significant association between tooth brushing and the prevalence of both *E. gingivalis* and *T. tenax* in both the ID (p<0.001) and non-ID groups (p<0.001). However, there was no significant relationship between the use of mouthwash and the prevalence of both *E. gingivalis* and *T. tenax* in either the ID (p = 0.821) or non-ID groups (p = 0.431) (**Tables 3**, **4**). Multiple logistic regression analysis indicated that residing in urban areas (crude OR = 2.63, 95% CI: 1.08–6.81, p = 0.029), parental education (crude OR = 3.37, 95% CI: 2.154–5.170, p<0.001), monthly family income (crude OR = 2.194, 95% CI: 1.096–4.390, P = 0.026), and tooth brushing (crude OR = 2.482, 95% CI: 1.617–3.810, p<0.001) were identified as independent risk factors for oral cavity parasites. Additionally, the analysis revealed that residence (crude OR = 0.421, 95% CI: 0.226–0.783, p = 0.006), monthly family income (crude OR = 2.194, 95% CI: 1.096–4.390, p = 0.026), and tooth brushing (crude OR = 2.482, 95% CI: 1.617–3.810, p<0.001) were also independent risk factors for oral cavity parasites among the examined risk factors and socio-economic parameters.

**Table 3:** Frequency of oral cavity parasites in children with intellectual disability based on the demographic, socio-economic, and risk factors.

Variable	Parasites		P value Chi-Square	Crude OR	95%CI	P value
	Positive (No)	Negative (No.)				
Age						
≥10 yrs	72 (46.5)	86 (54.4)	0.212	1.549	1	–
<10 yrs	20 (35.1)	37 (64.9)	–	1	0.827–2.901	0.172
Gender						
Male	55 (45.1)	67 (54.9)	–	1	–	–
Female	37 (39.8)	56 (60.2)	0.488	0.805	0.464–1.391	0.437
Residence						
Rural	24 (30.8)	54 (69.2)	–	1	1	–
Urban	68 (49.6)	69 (50.4)	0.01	2.217	1.234–3.984	0.008*
Parent education						
<Diploma	66 (42.9)	88 (57.1)	–	1	1	–
≥Diploma	26 (42.6)	35 (57.4)	1	0.99	0.544–1.804	0.975
Head of household’s occupation						
Unemployed	7 (38.8)	11 (61.2)	–	1	1	0.612
Employee	22 (41.5)	31 (58.5)	–	1	1	
Freelancer jobs	63 (48.7)	81 (51.3)	0.522	0.894	0.454–1.462	
Monthly family income						
≤200 USD	68 (60.7)	44 (39.3)	0	2.856	1.434–3.796	0.000*
200–600	20 (23.0)	67 (57.0)	–	–	1	
≥600 USD	4 (25.0)	12 (75.0)	–	–	1	
Tooth brushing						
Yes	19 (25.7)	55 (74.3)	–	1	–	–
No	73 (51.8)	68 (48.2)	0	2.691	1.433–3.967	0.000*
Mouthwash						
Yes	6 (40.0)	9 (60.0)	–	1	–	–
No	86 (43.0)	114 (57.0)	1	0.884	0.303–2.57	0.821

*p < 0.05, difference was statistically significant. -, Not determined.

**Table 4:** Frequency of oral cavity parasites in healthy children (non- intellectual disability) based on the demographic, socio-economic, and risk factors.

Variable	Parasites		P value Chi-Square	Crude OR	*95%CI*	*P value*
	Positive (No)	Negative (No.)				
Age						
≥10 yrs	28 (19.3)	117 (80.7)	–	–	1	–
<10 yrs	14 (20.0)	56 (80.0)	0.984	1.05	0.827–2.901	0.918
Gender						
Male	26 (18.9)	111 (81.1)	–	1	1	–
Female	16 (20.5)	58 (79.5)	0.369	1.36	0.62–3.47	0.279
Residence						
Rural	7 (8.1)	80 (91.9)	–	1	1	–
Urban	35 (27.3)	93 (72.7)	0	3.33	2.12–5.12	0.000*
Parent education						
<Diploma	20 (20.6)	77 (79.4)	–	1	1	–
≥Diploma	22 (18.6)	96 (81.4)	0.519	0.884	0.573–1.364	0.586
Head of household’s occupation						
Unemployed	3 (23.1)	10 (61.2)	0.404	1.42	1	0.372
Employee	13 (18.8)	56 (81.2)	–	1	1	
Freelancer jobs	26 (19.5)	107 (80.5)	–	1	0.67–3.41	
Monthly family income						
≤200 USD	31 (26.1)	44 (39.3)	0	3.345	1.64–4.23	0.000*
200–600 USD	8 (11.4)	67 (57.0)	–	–	1	
≥600 USD	3 (11.5)	12 (75.0)	–	–	1	
Tooth brushing						
Yes	15 (11.9)	111 (88.1)	–	1	–	–
No	27 (30.3)	62 (69.7)	0	2.321	1.34–3.541	0.000*
Mouthwash						
Yes	33 (18.9)	142 (81.1)	–	1	–	–
No	9 (23.7)	30 (76.3)	0.17	0.523	0.23–1.31	0.431

*p < 0.05, difference was statistically significant. -, Not determined.

## 4 Discussion

Today, it has been proven that the maintaining optimal oral health is a critical aspect of overall well-being, especially for children, and is of particular importance for individuals with special health needs (Gift and Atchison, 1995). Since, children with intellectual disability often face challenges in maintaining proper oral hygiene due to their motor, sensory, and intellectual impairments, which can lead to compromised oral health; therefore, there is a need to enhance the oral health status of these populations and establish an effective system for administering preventive interventions. Here, we aimed to evaluate the prevalence of *Entamoeba gingivalis* and *Trichomonas tenax* among children with ID in Lorestan province, in Western Iran through parasitological and molecular methods.

The present study showed that the total prevalence of the *E. gingivalis and T. tenax* was 40.5% and 42.8% by microscopic and PCR methods, respectively. Among positive samples, 61.9% and 38.1% children were positive for *E. gingivalis* and *T. tenax*, respectively. In a study conducted by Sharifi et al. (2020) on 315 adolescents in Kerman province, Iran, it was found that 9.2% and 11.4% tested positive for *E. gingivalis* and *T. tenax* through culture and PCR methods, respectively (Sharifi et al., 2020). Additionally, Mehr et al. (2015) demonstrated that the prevalence of *T. tenax* among individuals with Down syndrome and periodontitis in Tabriz, Iran was 18.8% as determined by PCR analysis (Mehr et al., 2015). Azadbakht et al. (2023) reported that among hemodialysis patients in Lorestan province, Western Iran, the frequencies of *E. gingivalis* and *T. tenax* were 17.1% and 14.5%, respectively (Azadbakht et al., 2023). Furthermore, Kooshki et al. (2023) observed that in children with cancer from Lorestan, Iran, the prevalence of *E. gingivalis and T. tenax* was 25.5% and 31.1% based on microscopic and PCR methods, respectively (Kooshki et al., 2023). The difference in the prevalence of these oral cavity parasites are likely influenced by factors such as the study population, sample size, and methodology employed.

Based on the statistical analysis, there was no significant relationship among age, gender, and prevalence of *E. gingivalis* and *T. tenax* parasites in children without ID. It has been proven that women display more favorable attitudes towards dental appointments, possess higher levels of oral health knowledge, and exhibit superior oral health practices compared to males (Lipsky et al., 2021). However, our results showed that there was no significant relationship among gender and prevalence of *E. gingivalis* and *T. tenax* parasites in non-ID children. Studies also demonstrated that children under 12 years of age are more susceptible to oral and dental diseases due to less knowledge and education, lifestyle habits and more contact with infectious agents (Hazavehei et al., 2015). In the present study, although the prevalence of oral cavity parasites was higher in children less than 10 years old, this difference was not significant. Similarly, in the study conducted by Kooshki et al. (2023) showed that there was no considerable correlation between the gender and the prevalence of *E. gingivalis* and *T. tenax* parasites in children with cancer from Lorestan, Iran (Kooshki et al., 2023).

Although, previous investigations have indicated a positive correlation between the level of education attained by parents and the improved oral health outcomes observed in their children. This relationship is believed to stem from the fact that parents with higher educational levels tend to possess greater awareness, financial means, and healthcare opportunities essential for fostering optimal health in their offspring (Minervini et al., 2023). Our study revealed that there was a higher prevalence of oral cavity parasites in children whose parents had lower levels of education; however, no statistically significant difference was noted.

Previous studies showed higher prevalence of oral and dental diseases in lower socioeconomic status may be due to lack of prevention and treatment services as well as poor diet high in sugar (Steele et al., 2015). On the other hand, children from higher income households have more chances to access dental care, including a more specific diagnostic assessment and have one or more filled teeth, explains some of the differences in oral and dental health due to socioeconomic status (Steele et al., 2015). In the present study although no correlation was observed among between head of household’s occupation and frequency of oral cavity parasites in children with ID; however, there a significant association was reported among the monthly family income and frequency of oral cavity parasites in children with ID.

Our results indicated that residing in urban areas and tooth brushing were identified as independent risk factors for oral cavity parasites in children with ID. In line with our results, Azadbakht et al. (2023) reported a significant association between brushing teeth and prevalence of oral protozoa in hemodialysis participants (Azadbakht et al., 2023). Another study conducted by Azadbakht et al. (2022) showed that there was a significant abscission among brushing teeth and the prevalence of oral cavity parasites in pregnant women in Lorestan province, western Iran; whereas no significant correlation was found by age, education, and use of mouthwash (Kooshki et al., 2023). Conversely, Kooshki et al. (2023) have reported that the living in urban regions were considerably linked with the prevalence of oral cavity parasites in children with cancer in Lorestan province, Iran (Kooshki et al., 2023).

Although this study has limitations such as a small sample size, it is noteworthy as it is the first study in the world to demonstrate the significant prevalence of oral cavity parasites in children with mental retardation. Hence, it is imperative for dental professionals and healthcare providers involved in monitoring health to possess knowledge of oral cavity parasites and their risk factors. This knowledge is essential for effectively identifying and addressing oral health concerns in children with intellectual disabilities. This proactive approach is essential for preventing or reducing potential periodontal and other oral complications that may arise in children with intellectual disabilities.

## 5 Conclusion

The study identified a notable prevalence of oral cavity parasites in children with intellectual disability in Lorestan province, Western Iran. It is imperative to recognize the primary risk factors and related socio-economic factors associated with these parasites, particularly inadequate teeth brushing, in order to enhance public and oral health strategies for children with intellectual disability. Therefore, pediatric dental professionals should remain vigilant regarding these risk factors to effectively recognize and address oral health issues in this population, thereby mitigating the occurrence of oral diseases and infections.

## Data availability statement

All data generated or analyzed during this study are included in this published article.

## Ethics statement

The studies involving humans were approved by The Ethical Committee of Lorestan University of Medical Sciences approved the study protocol under the No. of IR.LUMS.REC.1401.064. The studies were conducted in accordance with the local legislation and institutional requirements. Written informed consent for participation in this study was provided by the participants’ legal guardians/next of kin.

## Author contributions

BS: Conceptualization, Writing – review & editing. HM: Investigation, Methodology, Writing – review & editing. AK: Validation, Visualization, Writing – review & editing. FF: Investigation, Methodology, Writing – review & editing. FK: Supervision, Validation, Writing – original draft. PB: Data curation, Supervision, Formal analysis, Project administration, Software, Writing – review & editing.
